# [Retracted] Matrix metalloproteinase 14 participates in corneal lymphangiogenesis through the VEGF‑C/VEGFR‑3 signaling pathway 

**DOI:** 10.3892/etm.2025.12809

**Published:** 2025-01-27

**Authors:** Hai-Tao Du, Ping Liu

Exp Ther Med 12:2120–2128, 2016; DOI: 10.3892/etm.2016.3601

Following the publication of the above paper, a concerned reader drew to the Editor’s attention that, regarding the immunofluorescence microscopic assay experiments shown in Fig. 4A-C on p. 2125, all three panels contained overlapping sections of data, such that data from the same original source had apparently been used in the panels of this figure to portray the results of supposedly differently performed experiments. In addition, there were identical sections of data shown for the immunohistochemical staining of corneal tissue in Fig. 1D on p. 2123, which were difficult to attribute purely to coincidence.

Following an internal enquiry, the Editor of *Experimental and Therapeutic Medicine* has been able to verify the claims made by the interested reader; therefore, in view of the abovementioned anomalies that were identified, and owing to an overall lack of confidence in the presented data, the Editor has decided that the paper should be retracted from the Journal. The authors were asked for an explanation to account for these concerns, but the Editorial Office did not receive a reply. The Editor apologizes to the readership of the Journal for any inconvenience caused.

